# Mn^2+^-ZnSe/ZnS@SiO_2_ Nanoparticles for Turn-on Luminescence Thiol Detection

**DOI:** 10.3390/jfb8030036

**Published:** 2017-08-23

**Authors:** Mohammad S. Yazdanparast, William R. Jeffries, Eric R. Gray, Emily J. McLaurin

**Affiliations:** 1 Department of Chemistry, Kansas State University, Manhattan, KS 66506, USA; yazdanparast@ksu.edu; 2 Department of Chemistry, Viterbo University, La Crosse, WI 54601, USA; wjeffr59412@viterbo.edu; 3 Department of Chemical Engineering, Kansas State University, Manhattan, KS 66506, USA; ericgray24@ksu.edu

**Keywords:** sensing, doped nanocrystals, luminescence, phosphorescence, thiols

## Abstract

Biological thiols are antioxidants essential for the prevention of disease. For example, low levels of the tripeptide glutathione are associated with heart disease, cancer, and dementia. Mn^2+^-doped wide bandgap semiconductor nanocrystals exhibit luminescence and magnetic properties that make them attractive for bimodal imaging. We found that these nanocrystals and silica-encapsulated nanoparticle derivatives exhibit enhanced luminescence in the presence of thiols in both organic solvent and aqueous solution. The key to using these nanocrystals as sensors is control over their surfaces. The addition of a ZnS barrier layer or shell produces more stable nanocrystals that are isolated from their surroundings, and luminescence enhancement is only observed with thinner, intermediate shells. Tunability is demonstrated with dodecanethiol and sensitivities decrease with thin, medium, and thick shells. Turn-on nanoprobe luminescence is also generated by several biological thiols, including glutathione, *N*-acetylcysteine, cysteine, and dithiothreitol. Nanoparticles prepared with different ZnS shell thicknesses demonstrated varying sensitivity to glutathione, which allows for the tuning of particle sensitivity without optimization. The small photoluminescence response to control amino acids and salts indicates selectivity for thiols. Preliminary magnetic measurements highlight the challenge of optimizing sensors for different imaging modalities. In this work, we assess the prospects of using these nanoparticles as luminescent turn-on thiol sensors and for MRI.

## 1. Introduction

Imaging of biological environments is a critical strategy for the discovery and treatment of disease [1,2,3]. The desire to resolve microscopic events spatially and temporally has led to a multitude of imaging probes, which provide advances in resolution, sensitivity, targeting, and treatment [4,5,6,7]. Despite these advances, no single probe possesses all the properties necessary for comprehensive diagnostics. This limitation sparked an interest in combining imaging modalities to take advantage of their complementary abilities [8,9]. For example, good spatial resolution achieved by magnetic resonance imaging (MRI) may be combined with the high sensitivity of optical imaging [8]. Then, MRI can pinpoint the position of a probe, and optical imaging can yield detailed information on the probe’s local environment. Development of new and improved multimodal probes is an active area of interdisciplinary research, and advances make earlier identification and real-time monitoring of various diseases possible. 

Probes for optical imaging must be emissive, and preferably be photostable and absorb light at wavelengths in the near-infrared (NIR), where there is minimal absorption by tissue [7,10,11]. Semiconductor nanocrystals (NCs) are popular due to their photostability, good emission quantum yields, and large two-photon absorption cross-sections, which provide a pathway for NIR excitation of these materials, which usually only absorb at higher energies. They also provide a scaffold for the construction of nanoprobes [3,11,12,13], and are used in the construction of magneto-optical imaging agents in several ways [8]. One of the simplest, both in design and synthesis, is the incorporation of paramagnetic Mn^2+^ in a NC matrix [14,15,16]. This design provides the benefits of co-localization and tunability by changing the Mn^2+^ concentration and matrix. 

Contrast agents enhance resolution in MRI by providing a faster relaxation pathway for the nuclei of interest, which are usually water protons. The longitudinal and transverse relaxation rates (*T*_1_ and *T*_2_ respectively) are generally defined by Equation 1:

(1) $$\frac{1}{{\left(T_{i}\right)}_{obs}}=\;\frac{1}{{\left(T_{i}\right)}_{d}}+r_{i}\left[M\right],\;i\;=\;1,\;2$$

here, the observed solvent relaxation rate, *T_obs_*, and the diamagnetic contribution to the relaxation rate, *T_d_*, are linearly related to the concentration of paramagnetic species, *M*. The efficiency with which the complex enhances proton relaxation rates, the relaxivity (*r_i_*), is commonly used for comparison of different contrast probes. The relaxivity can be determined from the slope of a plot of the inverse of the observed relaxation rate versus the concentration of probe. As shorter *T*_1_ values lead to greater image intensities, larger values for *r*_1_ are generally desired. 

Optical sensors are available for detection of many analytes, and the high functionality of thiols within cells creates an immediate demand for the development of suitable probes for their continuous detection [17,18,19,20]. The literature describes multiple detection methods for thiols and thiol-containing peptides, but most of the available probes are based on organic dyes, which are prone to photobleaching, produce broad photoluminescence (PL) spectra, and are reliant on cellular extracts [21,22]. Atypical cellular thiol levels can lead to heart disease, cancer, stroke, and many neurological disorders [23,24,25,26,27,28,29]. One of the most abundant cellular thiols is the cysteine-containing tripeptide glutathione (GSH). GSH is a vital antioxidant for detoxification of reactive oxygen species, and patients with dementia often have low concentrations of GSH within the brain [22,26]. Intracellular GSH concentrations range from 1 to 15 mM, with poorly established concentrations across cellular compartments [30,31]. The development of probes that are resistant to photobleaching and provide real-time monitoring of thiols is of practical importance.

**Figure 1:**
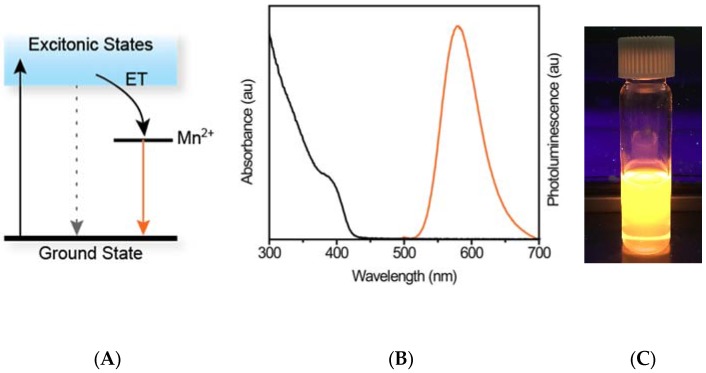
(**A**) Energy level diagram describing the Mn^2+^ luminescence process. Following excitation from the ground state to the excitonic states, energy is rapidly transferred to the Mn^2+^ ion. The resulting luminescence is due to the ^4^T_1_ → ^6^A_1_ spin-forbidden transition, and is therefore long-lived. (**B**) UV-vis absorption and photoluminescence (PL) spectra of Mn^2+^:ZnSe nanocrystals (NCs) illustrating the large Stokes shift present in these materials. (**C**) Photograph of a solution of orange-emitting Mn^2+^:ZnSe/ZnS NCs under UV light.

Mn^2+^-doped wide bandgap semiconductors are used in many photo- and electroluminescent applications [32,33,34], and the availability of luminescent colloidal NCs of these materials facilitated expanding their use to bioimaging and sensing [35,36,37]. Figure 1a illustrates an energy level diagram for Mn^2+^-doped wide bandgap semiconductors. After excitation (black arrow), energy is rapidly transferred (ET) to the Mn^2+^ ^4^T_1_ excited state, and then relaxes to the ^6^A_1_ ground state (orange arrow). This energy is released radiatively, producing a peak centred near 590 nm. Representative UV-vis absorption and PL spectra of Mn^2+^:ZnSe NCs in toluene are shown in Figure 1b, and a photograph of the NCs under UV light (Figure 1c) illustrates their bright, orange luminescence. To preserve this luminescence in aqueous solution, a thick shell of ZnS is often added to the NC. This shell passivates the surface of the NCs and isolates them from their surrounding environment [38]. For applications in which interaction between the NC and its surroundings are desired, such as sensing and MRI, the use of shells must be balanced with the desired degree of NC-local environment contact. In addition, for Mn^2+^-doped wide bandgap NC magneto-optical imaging agents, there is a trade-off between luminescence quantum yield (QY) and Mn^2+^ concentration [39,40,41,42]. At high dopant concentrations, which are useful for magnetic imaging, the luminescence is quenched [43].

This paper investigates water-soluble Mn^2+^:ZnSe/ZnS@SiO_2_ nanoparticles (NPs) with tunable, turn-on luminescence in the presence of biological thiols. Our goal of sensor design through surface modification expands opportunities for new probes and sensing modalities. Specifically, NP probes with tunable sensitivities to biologically relevant thiols provide a platform for the investigation of the effects of ZnS barrier layers and SiO_2_ encapsulation on the luminescent and magnetic properties of Mn^2+^-doped wide bandgap semiconductor NCs. Other semiconductor NC thiol sensors with turn-on luminescence employ NCs with thick protecting layers, so thiol sensitization requires external probes and quenchers [44,45,46,47,48,49]. In fact, thiols are well-known to quench NC PL [50] with few exceptions. Nearly a decade ago, Pradhan et al. reported enhancement of Mn^2+^ PL in Mn:ZnSe/ZnSe core/shell NCs in the presence of thiol [51]. Mercaptopropionic acid increased the luminescence of NCs with medium ZnSe shells, but the NCs were unstable in air and room light. Here, we show Mn^2+^-doped NCs and NPs exhibit turn-on luminescence in the presence of several thiols. By changing the NC surface through addition of ZnS, the degree of interaction between thiol and NP can be controlled. These NPs have tunable sensitivities corresponding to the ZnS shell thickness.

## 2. Results and Discussion

To explore the potential application of these Mn^2+^-doped NCs as sensors, the luminescence of these NCs in the presence of thiol was studied. Of particular interest is the possibility of tuning the probe sensitivity using ZnS barrier layers. This prospect was first examined using Mn^2+^:ZnSe core NCs in organic solvent. We impose NC luminescence sensitive to thiols by mitigating the NC–thiol interaction. Mitigation is achieved by varying the NC shell density and the corresponding luminescence change is caused by direct interaction between the NCs and thiol.

Our NP sensors are synthesized in a multistep process described in detail in the methods section. Briefly, luminescent Mn^2+^-doped core NCs form from isothermal decomposition of a single-source precursor, as described previously [14,52]. The reaction is monitored using UV-vis absorption, during which a first-absorption feature, corresponding to the ZnSe quantum dots, grows in near 380 nm and redshifts as the NCs increase in size. The small amount of Mn^2+^ precursor added prevents formation of MnSe, and the product core NCs exhibit properties corresponding mostly to their ZnSe lattice, with the large exception of the PL, which corresponds to the Mn^2+^ ^4^T_1_ → ^6^A_1_ transition observed near 590 nm (Figure 1b). The amount of Mn^2+^ was adjusted to provide bright PL and limit oxidation after exposure of the core NCs to air. 

After core synthesis and isolation, shells of ZnS are added to the NCs using an adapted process [53,54]. These shells serve to both passivate surface defects and isolate the NC core from its surrounding environment. More isolation and surface passivation decreases the sensitivity of the NC to its local environment. For sensor design, this is key in tuning sensitivity. Using an adaptation of the well-established selective ion adsorption and reaction (SILAR) method, monolayers (MLs) of Zn^2+^ and S^2−^ are added to the Mn^2+^:ZnSe core NCs. To examine the effect of shell thickness on thiol sensitivity, samples with thin (~1–2 MLs), medium (~4 MLs) and thick (~8–10 MLs) ZnS shells were prepared and used in all further experiments. Shell thicknesses are estimated using the previously established parameters for SILAR, and are not directly measured. Figure S1 features TEM images of the NCs with and without ZnS shells. The addition of thin shells primarily passivates the NC surface with little isolation from its surroundings. Surface defects arise through the purification process, during which the Mn^2+^:ZnSe core NCs are exposed to oxidizers, both in air and in solvent. 

### 2.1. Shell-Thickness Dependent Thiol Detection in Organic Solvent

**Figure 2:**
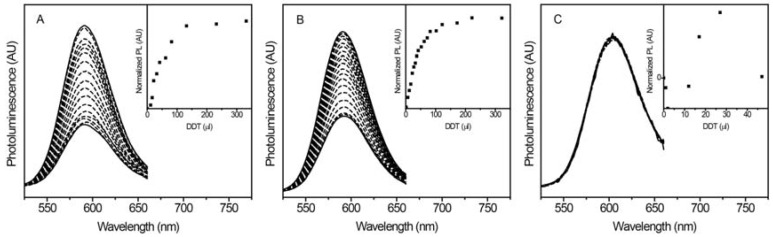
PL spectra of Mn^2+^:ZnSe/ZnS NCs suspended in chloroform with: (**A**) thin, (**B**) medium, and (**C**) thick ZnS shells in the presence of increasing amounts of dodecanethiol (DDT). Insets: corresponding plots of normalized PL (PL change) with increasing thiol. The increase in PL in the presence of DDT is large for samples with medium shells and thin shells, whereas the change in PL in the thick shell sample is negligible.

The luminescence response of the NCs to thiol was first tested in organic solvent. Following isolation from the reaction mixture, the NCs are soluble only in hydrophobic environments. The hydrophobic dodecanethiol (DDT) was used to test the thin shell NC response. As seen in Figure 2a, there is a large increase in Mn^2+^ luminescence with increasing amounts of DDT. A similar increase is observed when a medium thickness shell is added (Figure 2b), but there is only a negligible change for the thick shell sample (Figure 2c). Scatter plots of the PL intensity vs thiol show initial increases for thin (Figure 2a inset) and medium (Figure 2b inset) shell samples, but no change for the thick shell sample (Figure 2c inset). The scatter plot in Figure 2a shows an increase in PL up to ~75 μL DDT before saturating, and the medium shell sample (Figure 2b inset) shows an increase in PL up to ~150 μL DDT before leveling out. This difference is also observed when comparing sensitivities for similar sensors. As shell thickness increased, the sensitivity first increased, followed by a large decrease from medium to thick, indicating that these NPs are intrinsically less responsive. Although sample-to-sample variations yielded different sensitivities, this trend was reproducible for thin, medium, and thick shell samples, illustrating the tunability of the NC sensitivity using ZnS barrier layers in organic solvent. 

### 2.2. Thiol Sensing in Aqeuous Solution

To use these probes for sensing biological thiols, they must be transferred to aqueous solution. Methods for phase transfer such as simple ligand exchange were unsuccessful, as the particles tended to aggregate and fall out of solution. The method that generated the best sensors was the addition of a silica shell to the core/shell NCs using an adapted inverse micelle approach detailed in the methods section [55]. The product NPs consist of a Mn^2+^-doped ZnSe core with shells of ZnS and a coating or encapsulating layer of silica, which provides water dispersibility. TEM images (Figure S2) indicate the particles are on the order of 200 nm in diameter and dynamic light scattering (DLS) measurements indicate hydrodynamic diameters of ~1000 nm (Figures S3,S4). The discrepancy between TEM size and DLS size is ascribed to swelling or aggregation of the particles in solution, but rapid decomposition of the NPs during TEM imaging was also observed, possibly decreasing the NP size.

#### 2.2.1. Luminescence Enhancement with Different Thiols

**Figure 3:**
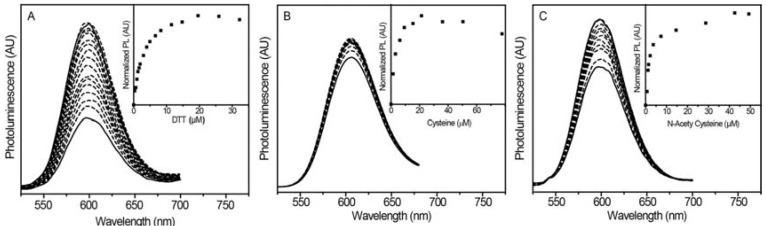
PL spectra of Mn^2+^:ZnSe/ZnS@SiO_2_ NPs with medium ZnS shells encapsulated in silica suspended in phosphate-buffered saline (PBS) with successive addition of 1 mM (**A**) dithiothreitol (DTT), (**B**) L-cysteine (CYS), and (**C**) N-acetylcysteine (NAC). Insets: corresponding plots of normalized PL (PL change) with increasing thiol.

Titrations of different thiols with these NPs were done in aqueous solution to characterize their specificity and scope. Four biologically relevant thiols were chosen for the luminescence titrations: dithiothreitol (DTT), N-acetylcysteine (NAC), cysteine (CYS), and glutathione (GSH). Aqueous solutions of these thiols were made and titrated into NP solutions. PL spectra from an exemplary titration are shown in Figure 3a. As the concentration of DTT increases from 0 to 20 µM, the NPs exhibit increasing Mn^2+^ PL. A scatter plot of PL intensity against [DTT] (Figure 3a inset) illustrates a linear increase in PL prior to a decrease at concentrations >20 µM. DLS characterization shows a small change in size after DTT addition, possibly due to a reduction in aggregation. Quantum yields were 2.5% without DTT, and 8.0% with DTT, even after several weeks, indicating long-lived luminescence enhancement in the presence of DTT.

Similar luminescence enhancement is observed with other thiols. Figure 3b,c show luminescence increasing with different sensitivities to CYS and NAC, respectively. For CYS, the scatter plot of PL intensity against [CYS] illustrates a linear increase prior to PL decreasing at concentrations >25 µM (Figure 3b inset). Similarly, for NAC, the scatter plot shows a linear PL increase prior to decreasing at [NAC] >40 µM (Figure 3c inset). In addition to exhibiting turn-on luminescence in the presence of several biologically relevant thiols, this enhancement extends to different luminescent materials, namely Mn^2+^:ZnCdSe/ZnS@SiO_2_ dual-emitting NPs of interest for optical thermometry. As shown in Figure S5, the PL of both peaks increases in the presence of DTT. This turn-on response is perpendicular to the ratiometric changes in dual-emission with temperature, and highlights the utility of this turn-on sensing mechanism with these NPs. 

#### 2.2.2. Glutathione Sensing with Different Shell Thicknesses

**Figure 4:**
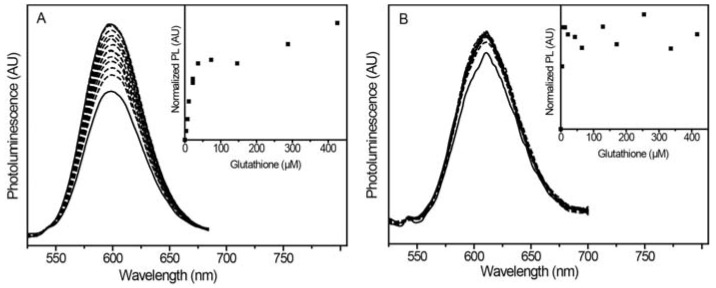
(**A**) PL spectra of Mn^2+^:ZnSe/ZnS@SiO_2_ NPs with medium ZnS shells suspended in PBS with successive addition of 10 mM L-glutathione (GSH). As the concentration of GSH increases from 0–25 µM, the Mn^2+^ PL also increases. (**B**) PL spectra of Mn^2+^:ZnSe/ZnS@SiO_2_ NPs with thick ZnS shells suspended in PBS with successive addition of 10 mM GSH. The Mn^2+^ PL increases as [GSH] approaches ~10 µM, and quickly levels off. Insets: corresponding plots of normalized PL (PL change) with increasing thiol. The medium shell sample shows linear PL restoration prior to plateauing at 37 µM. The thick shell sample shows nearly immediate stagnant PL response, with no significant change over a range of 9–420 µM.

In addition to the three thiols used in the previous section, the NPs also exhibited turn-on luminescence with GSH. To see if sensor tunability using shell thickness extends to aqueous solution, we prepared Mn^2+^:ZnSe/ZnS@SiO_2_ NPs with varying ZnS densities: thin, medium, and thick. The NPs with a thin ZnS shell did not suspend well in aqueous solution, and their PL was too low to obtain good signal-to-noise. Titrations of NPs with medium ZnS shells showed enhanced Mn^2+^ luminescence with increasing amounts of GSH (Figure 4a). Importantly, the probe luminescence was sensitive enough to differentiate GSH at concentrations of 0 to 22 μM (Figure 4a inset). To assess if the sensitivity is tunable by adjusting the ZnS shell thickness, aqueous GSH titrations were also performed with NPs consisting of a thick ZnS shell (Figure 4b). The inset of Figure 4b shows the PL intensity initially increased a small amount before rapidly leveling off. No significant intensity change was measured between concentrations ranging from 9 to 420 μM. This experiment supports the notion that increasing surface protection and passivation through ZnS shells reduces the NP sensitivity. 

#### 2.2.3. Thiol Selectivity

**Figure 5:**
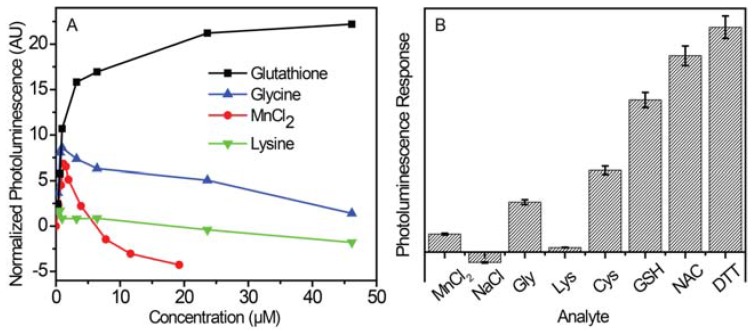
(**A**) Scatter plot of normalized Mn^2+^:ZnSe/ZnS@SiO_2_ PL vs analyte concentration for glutathione (GSH), glycine (GLY), MnCl_2_, and lysine (LYS). (**B**) PL response of NP solutions in the presence of various analytes. Relative intensities were obtained by averaging the PL response as a function of concentration. Multiple regression analysis results indicate that all control analyte PL intensities were significantly less than GSH, NAC, and DTT.

Although sensitivity to several analytes can be advantageous under some conditions, broad analyte enhancement of NP PL limits the utility of these sensors. For applications in biology, the specific environment of the sensor must be considered. For intracellular environments, the concentrations of GSH are heterogeneous, and the redox values for different molecules change depending on their microscopic environment [31,56]. Instead of examining strong reductants, we selected several salts and amino acids to assess possible issues with selectivity. This is not a comprehensive assay, but it does provide insight into sensitivities related to two key areas: 1) additional dopant (Mn^2+^) and other ions, and 2) molecules with functional groups, especially amines, known to strongly interact with nanoparticle surfaces. This investigation revealed that salts and amino acids did not induce sufficient optical emission (Figure 5). Figure 5a shows a scatter plot of the PL change of these NPs in the presence of GSH and several other analytes. Although the NP PL increases at first for both glycine (GLY) and MnCl_2_, a rapid decrease follows beyond a concentration of few μM. The PL spectrum of NPs and lysine (LYS) initially exhibits a small decrease before continuously decreasing at concentrations approaching 45 μM. PL spectra and corresponding scatter plots can be found in the SI (Figures S6–S9). Further analysis of this data and that for other analytes is plotted in Figure 5b. ANOVA and multiple regression variance tests indicate that the tested thiols produced stronger PL responses than control analytes. The best response was from DTT, which exhibited *p*-values <0.00026, and apart from GLY, NAC and GSH had *p*-value thresholds of 0.00032 and 0.0031, respectively. Table S1 gives the full list of values, which overall indicate that the signal response of the NPs is more sensitive towards thiols relative to the other salts and amino acids tested.

### 2.3. Prospects for Multimodal Imaging and Sensing

To assess the potential use of these Mn^2+^-containing NPs as bimodal imaging agents and sensors, we examined their magnetic properties in the presence and absence of DTT. Although the mechanism of thiol PL enhancement is not established, proposed pathways in other Mn-doped nanomaterials include reduction of surface defects, prevention of surface oxidation, and formation of an “S” shell [57]. PL enhancement by surface passivation was previously ascribed to Mn^3+^ reduction [58], but is not likely to be the only process occurring. One of the more puzzling aspects of the interaction of thiols with this type of NC is that luminescence enhancement occurs at all. Many other chalcogenide NCs (ZnSe, CdSe, and derivatives of these) lose luminescence with thiol, and the mechanism of these alternative pathways continues to be elusive [50,51,57,59,60]. Other effects due to cooperative thiol interactions or the changing NCs with increasing thiol may also be in play [61]. More recent studies suggest enhancement is due to reduction of surface traps with a broad distribution of potentials [62,63], and unlikely to perturb the Mn. In fact, the *T*_1_ values measured for these Mn^2+^:ZnSe/ZnS@SiO_2_ NPs (Table 1) are very similar for all NP samples despite the presence of thiol. Also evident from the table is the small difference between the relaxation times for the blank samples (water) and those of the samples with NPs. This indicates the effect of the NPs on relaxation time is small under these conditions. This is not surprising, as the concentrations of NPs needed for luminescence imaging are much smaller than those required for MR imaging. The low concentration of Mn^2+^ in the samples and the isolation of these cations from their surroundings by the shells and layers of other materials added would also decrease the effect of the NPs on relaxation time. Previously, particles with good relaxivity were obtained by Mn^2+^-doping of shell layers [15], or use of NCs without shells [16]. These results highlight the additional challenges in multimodal sensor design for luminescence and magnetism. Optimizing a sample for optical imaging and sensing of an analyte in a specific concentration range can limit the usefulness of the probe for magnetic measurements. These NPs designed for tunable thiol sensing are not optimized for use in magnetism, but the potential for multiparameter control provides an enticing opportunity for sensor design and development. 

## 3. Conclusions

Mn^2+^-doped NCs exhibit turn-on luminescence in the presence of thiols. Tunable sensitivity is obtained simply by changing the thickness of the ZnS barrier shell, which controls the ability of the thiol to interact with the NC surface. This behavior was first observed in organic solvent, and can be preserved in aqueous solution when a silica shell is added to the NCs. This is demonstrated by luminescence enhancement with several thiols of interest. The NPs are selective for thiols, but unable to distinguish between them. Optimization for a desired sensing range is possible for the concentrations and analytes included here. Mn^2+^-doped NPs have potential for multimodal imaging, although these probes, which are designed for tunable thiol sensing, must be optimized for use in MRI. Nevertheless, these NPs are stable within aqueous environments and superior to other optical sensing agents such as organic dyes. The potential for tunable control over luminescent and magnetic properties provides an attractive opportunity for multimodal sensor advancement. Further development hinges on successful deployment of these NPs in biological environments, and balancing magnetic and optical properties to mitigate decreases in sensitivity and brightness while still achieving enhanced relaxation times.

## 4. Materials and Methods 

All the chemicals used are commercially available and were used without further purification; Manganese(II) chloride tetrahydrate (99.99%), L-glutathione reduced (GSH, ≥98.0%), Sodium chloride (≥99%), oleylamine (OLA, ≥98%), dodecanethiol (DDT, ≥98%), selenium (≥99.5%), sulfur (≥99.5%), tetramethylammonium chloride (≥98%), triethylamine (TEA, ≥99%), octadecene (ODE, 90%), octylphenoxypolyethoxyethanol (IGEPAL CA-520, CA-630, CA-720), tetraethylorthosilicate (TEOS, 99.999%), ammonium hydroxide solution (7N), agarose, cyclohexane, toluene, water, phosphate-buffered saline (PBS), and chloroform from Aldrich, N-acetylcysteine (NAC, 98%), phenylselenol (PhSeH, 98%), cadmium chloride hemipentahydrate (≥99%), and methanol from Acros, zinc nitrate hexahydrate (≥99%) from J. T. Baker, acetone, dithiothreitol (DTT), glycine (GLY, ≥98.5%), cysteine (CYS, ≥99.5%), L-lysine (LYS, ≥98.5%), Fluorescein, and Rhodamine B from Fisher. 

### 4.1. [NMe_4_]_2_[Zn_4_(SePh)_10_] 

The cluster precursor was prepared by adapting previous methods [64,65]. Three separate solutions were prepared and degassed for 30–60 min by bubbling with N_2_: 1) Zn(NO_3_)_2_·6H_2_O (7.318 g) in 35 mL MeOH, 2) Me_4_NCl (4.42 g) in 20 mL MeOH, and 3) TEA (9.5 g) in 20 mL MeOH. Under a N_2_ overpressure, PhSeH (10 g) was added to solution 3, followed by stirring for 20 min. Solution 1 was then transferred to solution 3 dropwise via cannula, forming a cloudy solution. Next, solution 2 was cannula transferred to solution 3 over a period of 10–30 min, followed by cooling in an ice bath for 30 min. The solid was then filtered and rinsed thoroughly with MeOH and toluene, yielding a white powder.

### 4.2. Mn^2+^:ZnSe and Mn^2+^:ZnCdSe Nanocrystals

Colloidal Mn^2+^:ZnSe NCs were synthesized by adapting previously described methods [14,65]. Briefly, OLA (10 g), MnCl_2_·4H_2_O (0.0012 g, 6.1 μmol), and for the ZnCdSe alloys CdCl_2_·2.5H_2_O (0.0146 g, 64 μmol), were combined in a flask and degassed at 100 °C under vacuum for 120 min. After cooling below 80 °C, the cluster precursor (0.2 g, 0.1 mmol) and Se (0.0108 g, 0.137 mmol) were added under N_2_ overpressure. After briefly degassing the solution under vacuum at 100 °C, the reaction was heated to 280 °C under N_2_ and the NCs were grown at this temperature for 15 min. After cooling below 80 °C, the NCs were isolated by precipitation and resuspension with methanol and toluene, respectively.

### 4.3. ZnS Passivation Shell Growth 

For addition of zinc sulfide to the nanocrystal surfaces, a previously reported procedure was adapted [54]. First, core particles suspended in a small amount of toluene (about 25–50% of a core synthesis, above) were added to a 50 mL three-neck flask containing octadecene (ODE, 1.5 g) and oleylamine (OLA, 1.5 g). The reaction flask was kept under vacuum at 100 °C for 30 min. Under a nitrogen atmosphere, the reaction was heated to 220 °C, at which point an ODE solution containing zinc oleate (0.2 M) was added to the nanocrystal suspension over a period of 3–4 min by syringe. The zinc precursor was allowed to react for 25 min prior to the addition of the sulfur precursor. A solution of sulfur in ODE (ODE-S), formed by combining elemental sulfur (1 mmol) and ODE (5 mL), was added to the core solution over a period of 5 min, using a syringe pump. The precursors were allowed to react for 25 min prior to the addition of more zinc precursor. This process was repeated until the desired shell thickness was reached. Following synthesis, these nanocrystals were washed by repeated precipitation with ethanol and resuspension in toluene. 

### 4.4. Water-Soluble Mn^2+^:ZnSe Nanoparticles

A previous procedure was adapted to form silica-encapsulated NCs soluble in aqueous solution [55]. Core/shell NCs were washed by repeated precipitation with acetone and resuspension in chloroform. Silica shells were prepared by adding IGEPAL (1.3 mL, CA-520, CA-630 or CA-720) to glass vials containing cyclohexane (10 mL). This solution was stirred for 30 min before 200 μL of dispersed NCs were added. After stirring for 15 min, TEOS (80 μL) was added and the solution was stirred for 10 min. Finally, NH_4_OH solution (150 μL) was added and samples were stirred overnight. 

### 4.5. Agarose Gel Protocol

A uniform solution of 0.05 g agarose in 5 mL water was prepared using a hot bath and then after cooling the solution to about 70 °C, 1 mL of aqueous solution of desired NPs was added to it and mixed thoroughly. The mixture was sonicated until an air-bubble free gel formed. 

### 4.6. Physical Measurements

In this study, a Cary 5000 UV-vis-NIR spectrophotometer was used for recording UV-vis absorption spectra. A PTI Quanta Master 400 fluorometer was used to record PL spectra, quantum yields, and perform thiol titration experiments. The samples were prepared in a cuvette with a stir bar. A syringe pump was used for the titration, with injection of thiol via a syringe as described previously [66]. While stirring, spectra were taken at intervals of 2–30 min, depending on sample brightness and sensitivity. Titrations were done until luminescence saturation or decrease. Relative quantum yields of samples, Φ_sam_, were calculated using Fluorescein and Rhodamine B in 0.1 N NaOH or water as the reference according to Equation 2:

(2) $$\Phi_{\mathrm{sam}}=\Phi_{\mathrm{ref}}\left(\frac{A_{\mathrm{ref}}}{A_{\mathrm{sam}}}\right)\left(\frac{I_{\mathrm{sam}}}{I_{\mathrm{ref}}}\right){\left(\frac{\eta_{\mathrm{sam}}}{\eta_{\mathrm{ref}}}\right)}^{2}$$

*A* is the measured absorbance, *η* is the refractive index of the solvent, *I* is the integrated photoluminescence, and Φ_ref_ is the emission quantum yield of the reference. Φ_ref_ was taken to be 0.95 for Fluorescein in 0.1 N NaOH, and 0.31 for Rhodamine B in water [67,68]. DLS measurements were performed using ZetaPALS (Brookhaven Instruments Corp., Holtsville, NY, USA). For MRI measurements, a sample of Mn^2+^:ZnSe/ZnS@SiO_2_ was diluted in 5 mL distilled water and divided into two tubes. DTT (0.0224 g, 0.15 mmol) was added to one of the tubes. The longitudinal relaxation time of these samples was determined using an inversion recovery pulse sequence using a 14.1 T NMR system (Bruker Avance III, WB, 600 MHz NMR-MRI). For TEM analysis, a drop of sample was dried on a copper grid. TEM images were recorded using an FEI Tecnai G2 Spirit BioTWIN microscope.

## Figures and Tables

**Table 1:** *T*_1_ values for medium shell Mn^2+^:ZnSe/ZnS@SiO_2_ NPs before and after thiol addition.

Run	NCs (s)	+DTT (s)	Water (s)
1	2.54888	2.63426	2.60044
2	2.53549	2.71390	2.69495
3	2.59246	2.64803	2.59054
4	2.63351	2.64220	2.61833

## Supplementary Materials

The following are available online at http://www.mdpi.com/2079-4983/8/3/36/s1, Figure S1: TEM of Mn^2+^:ZnSe and Mn^2+^:ZnSe/ZnS nanocrystals, Figure S2: TEM of Mn^2+^:ZnSe/ZnS@SiO_2_ nanoparticles, Figure S3: DLS of Mn^2+^:ZnSe/ZnS@SiO_2_ nanoparticles, Figure S4: DLS of Mn^2+^:ZnSe/ZnS@SiO_2_ nanoparticles + DTT, Figure S5: Titration of Mn^2+^:ZnCdSe/ZnS@SiO_2_ NPs with DTT, Figure S6: Titration of Mn^2+^:ZnSe/ZnS@SiO_2_ NPs with NaCl, Figure S7: Titration of Mn^2+^:ZnSe/ZnS@SiO_2_ NPs with LYS, Figure S8: Titration of Mn^2+^:ZnSe/ZnS@SiO_2_ NPs with GLY, Figure S9: Titration of Mn^2+^:ZnSe/ZnS@SiO_2_ NPs with MnCl_2_, Table S1: *p*-values for thiols and control analytes.
