# Supplementary material for: Mn2+-ZnSe/ZnS@SiO2 Nanoparticles for Turn-on Luminescence Thiol Detection
Source: J Funct Biomater. 2017 Aug 23;8(3):36. doi: 10.3390/jfb8030036 (PMC5618287; doi:10.3390/jfb8030036)
Supplement: Supplementary file 1 [file jfb-08-00036-s001.docx]

**Supporting Information**

**Mn^2+^-ZnSe/ZnS@SiO_2_ Nanoparticles for Turn-on Luminescence Thiol Detection**

**Mohammad S. Yazdanparast^1^, William R. Jeffries^2^, Eric R. Gray^3^, and Emily J. McLaurin^1,^***

*1. Department of Chemistry, 213 Chemistry and Biochemistry Building, 1212 Mid-campus Drive North, Kansas State University, Manhattan, KS 66506 United States*

*2. Department of Chemistry, 900 Viterbo Drive, Viterbo University, La Crosse, WI 54601 United States*

*3. Department of Chemical Engineering, 1005 Durland Hall, 1701A Platt St, Kansas State University, Manhattan, KS 66506 United States*

*mclaurin@ksu.edu

| *Index* |  | *Page* |
| --- | --- | --- |
| **Figure S1.** | TEM of Mn^2+^:ZnSe and Mn^2+^:ZnSe/ZnS nanocrystals | S2 |
| **Figure S2.** | TEM of Mn^2+^:ZnSe/ZnS@SiO_2_ nanoparticles | S2 |
| **Figure S3.** | DLS of Mn^2+^:ZnSe/ZnS@SiO_2_ nanoparticles | S3 |
| **Figure S4.** | DLS of Mn^2+^:ZnSe/ZnS@SiO_2_ nanoparticles + DTT | S4 |
| **Figure S5.** | Titration of Mn^2+^:ZnCdSe/ZnS@SiO_2_ NPs with dithiothreitol | S5 |
| **Figure S6.** | Titration of Mn^2+^:ZnSe/ZnS@SiO_2_ NPs with NaCl | S5 |
| **Figure S7.** | Titration of Mn^2+^:ZnSe/ZnS@SiO_2_ NPs with lysine | S6 |
| **Figure S8.** | Titration of Mn^2+^:ZnSe/ZnS@SiO_2_ NPs with glycine | S6 |
| **Figure S9.** | Titration of Mn^2+^:ZnSe/ZnS@SiO_2_ NPs with MnCl_2_ | S7 |
| **Table S1.** | *p*-values for thiols and control analytes | S7 |
|  |  |  |





50 nm

**A**





50 nm

20 nm

**B**







**D**

**C**

**Figure S1:** TEM images of Mn^2+^:ZnSe core (A) and Mn^2+^:ZnSe/ZnS core/shell nanocrystals with medium (B) and thick (C, D) shell thicknesses. Here, the shell thickness is based on the amounts of Zn^2+^ and S^2-^ added during the SILAR shell growth process.







**Figure S2:** TEM images of Mn^2+^:ZnSe/ZnS@SiO_2_ nanoparticles.


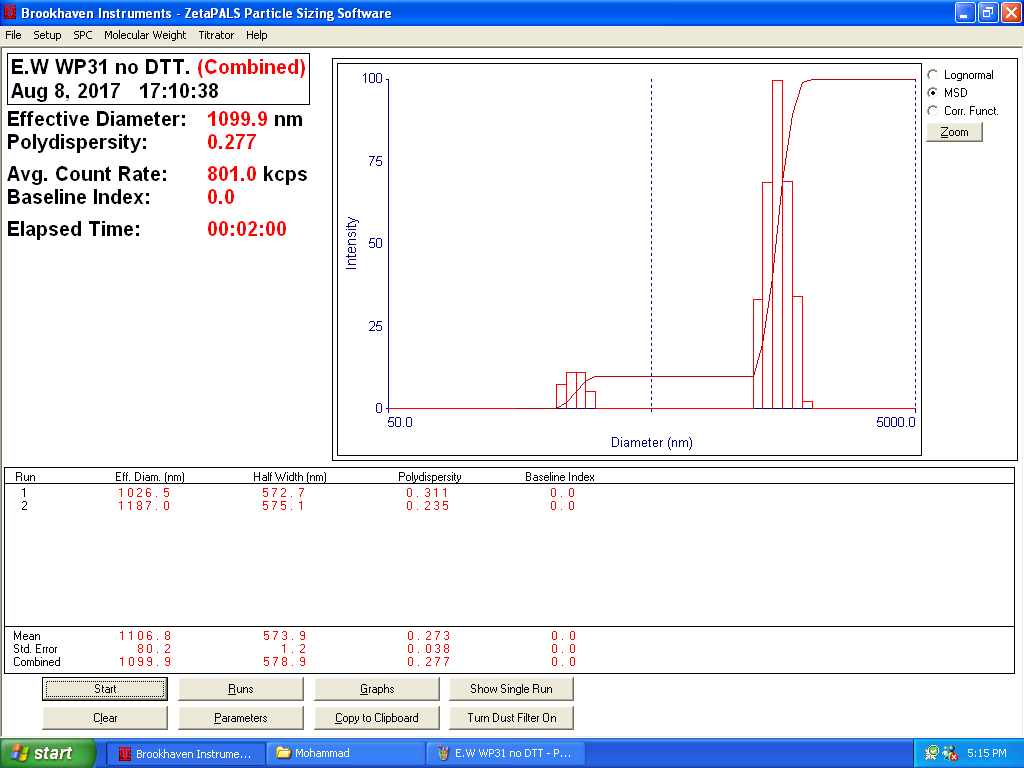


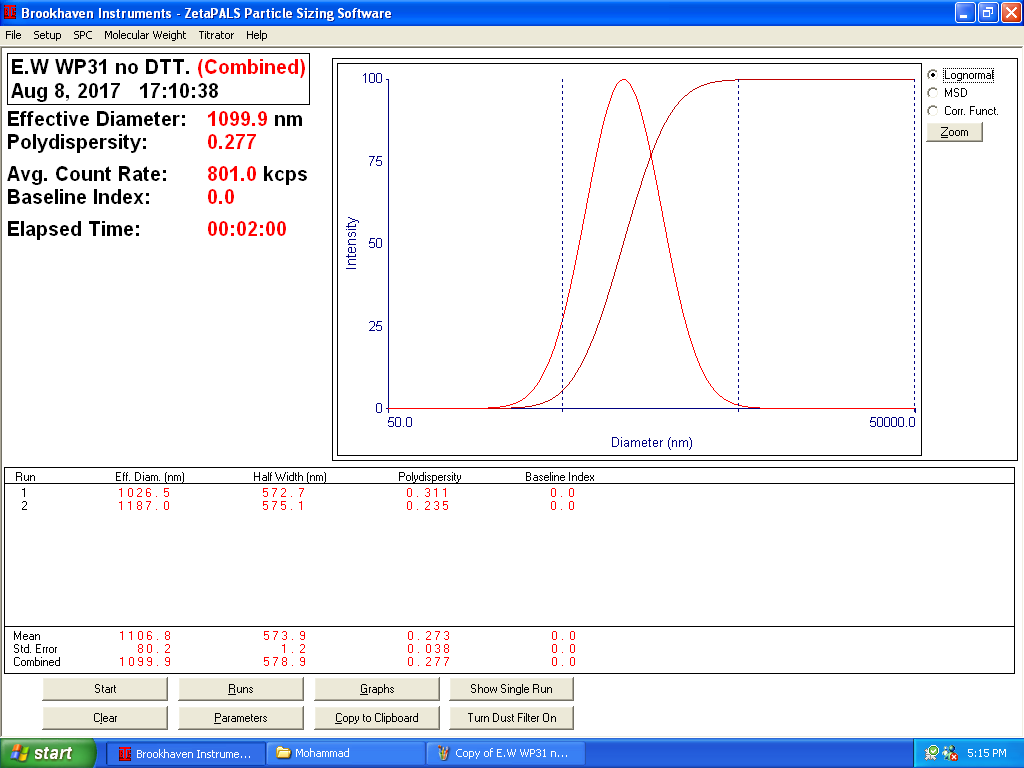


**Figure S3:** DLS characterization of Mn^2+^:ZnSe/ZnS@SiO_2_ nanoparticles.


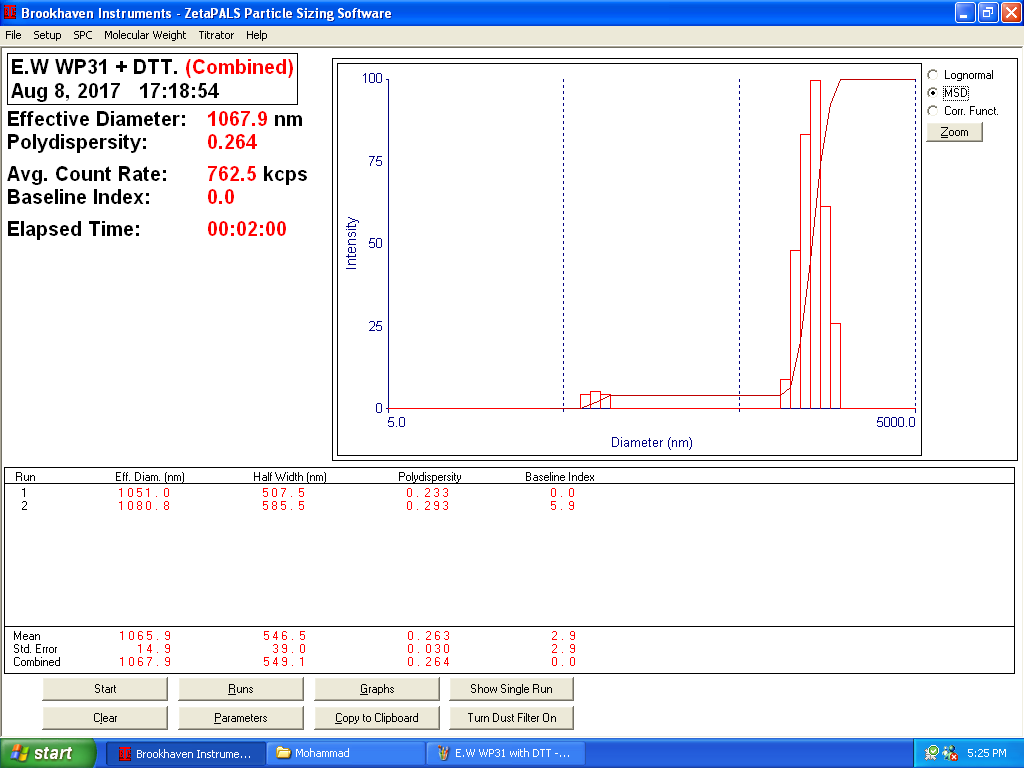


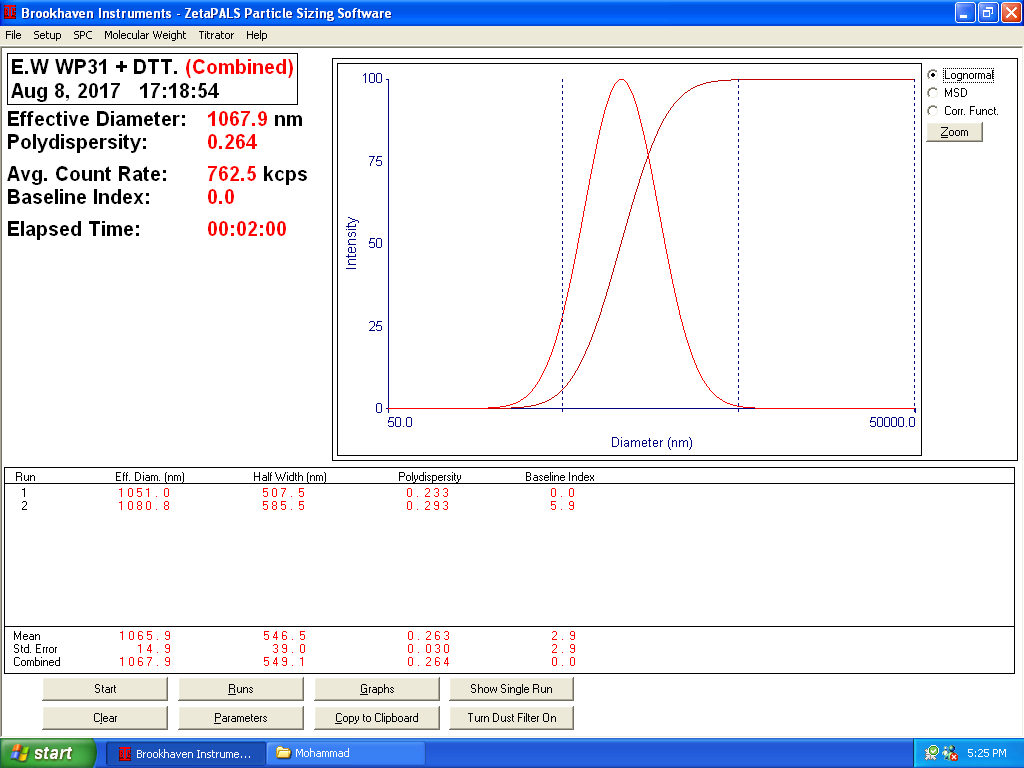


**Figure S4:** DLS characterization of Mn^2+^:ZnSe/ZnS@SiO_2_ nanoparticles after dithiothreitol addition.


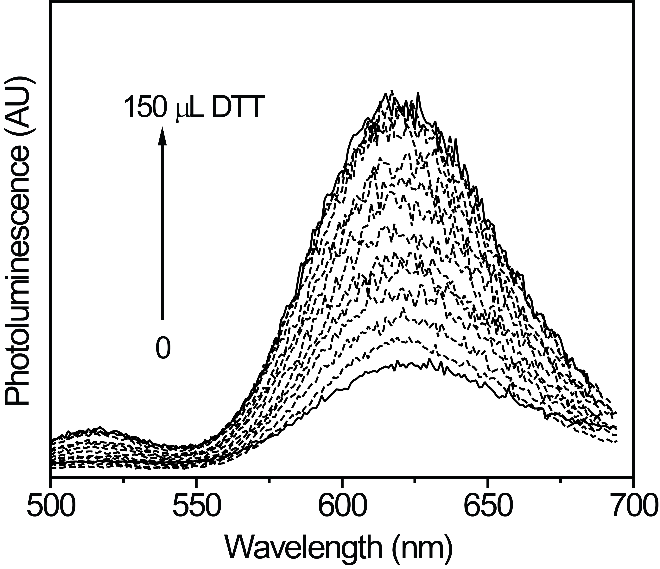


**Figure S5:** Photoluminescence spectra of Mn^2+^-doped ZnCdSe NPs with medium ZnS shells encapsulated in silica suspended in PBS with successive addition of 1 mM dithiothreitol (DTT).

**
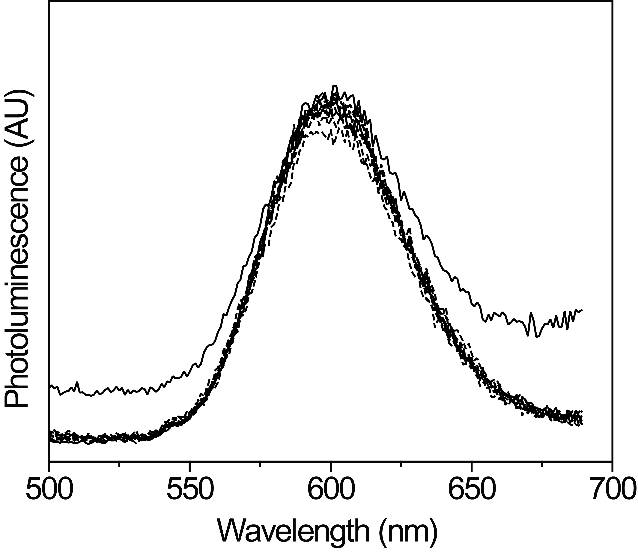

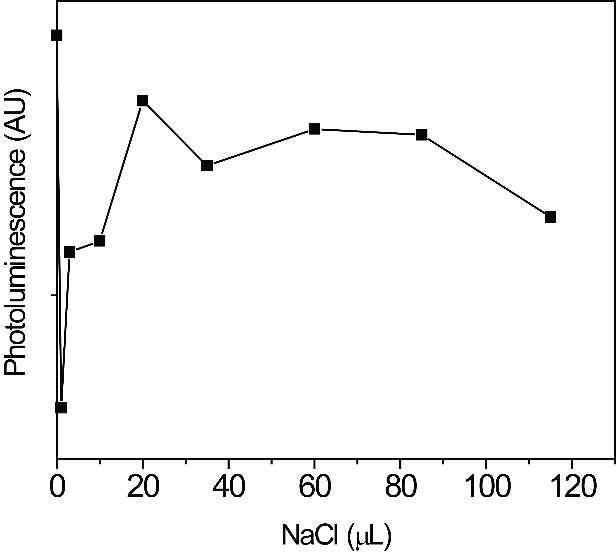
**

**Figure S6:** Left: Photoluminescence spectra of Mn^2+^:ZnSe/ZnS@SiO_2_ NPs with medium ZnS shells encapsulated in silica suspended in PBS with successive addition of 1 mM NaCl. Right: Corresponding scatter plot.


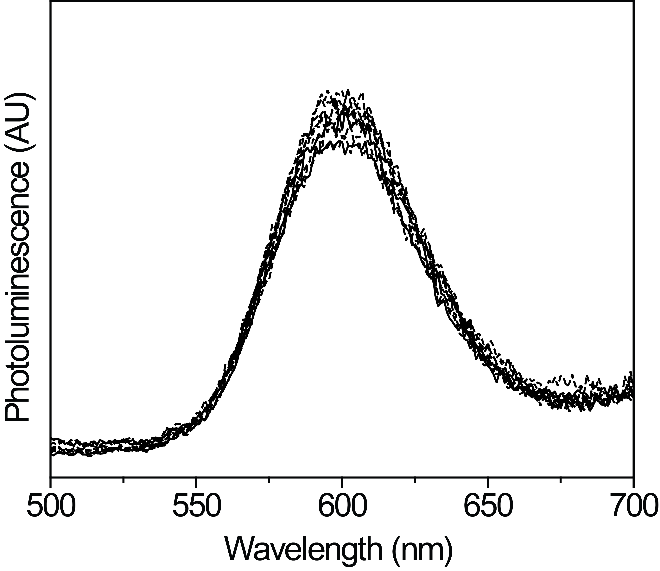

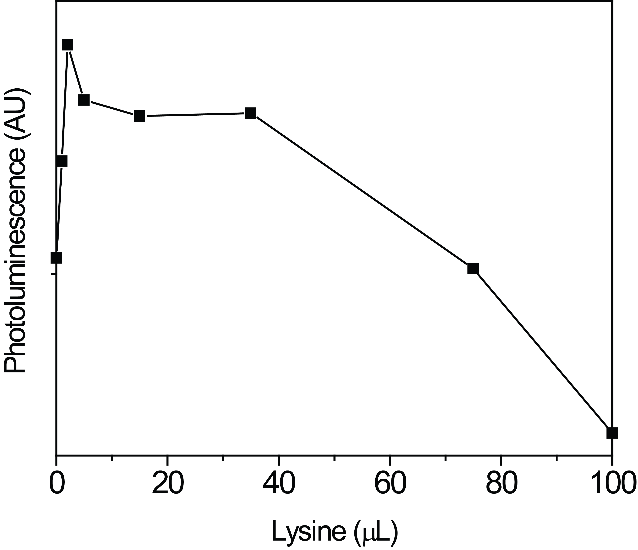


**Figure S7:** Left: Photoluminescence spectra of Mn^2+^:ZnSe/ZnS@SiO_2_ NPs with medium ZnS shells encapsulated in silica suspended in PBS with successive addition of 1 mM lysine (LYS). Right: Corresponding scatter plot.


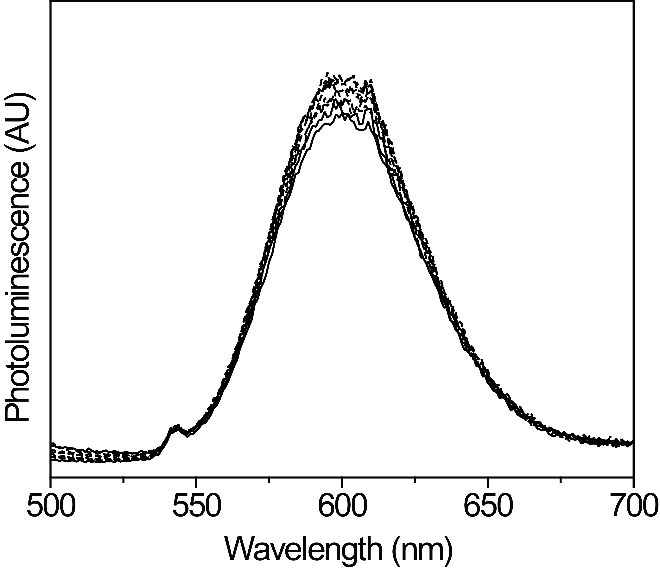

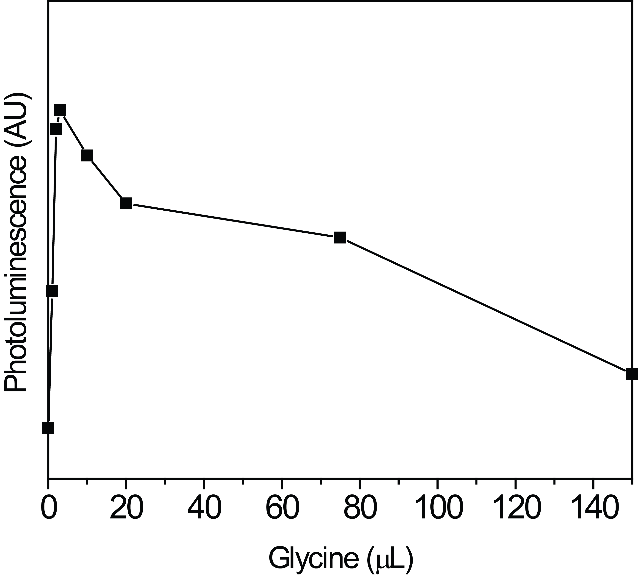


**Figure S8:** Left: Photoluminescence spectra of Mn^2+^:ZnSe/ZnS@SiO_2_ NPs with medium ZnS shells encapsulated in silica suspended in PBS with successive addition of 1 mM glycine (GLY). Right: Corresponding scatter plot.


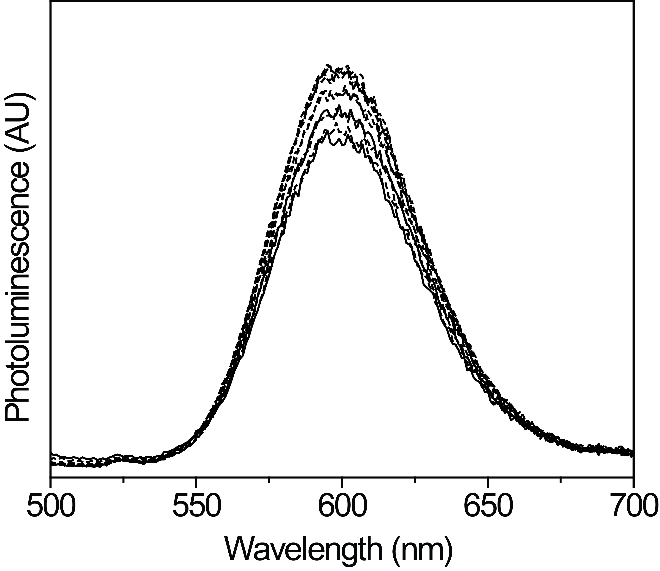

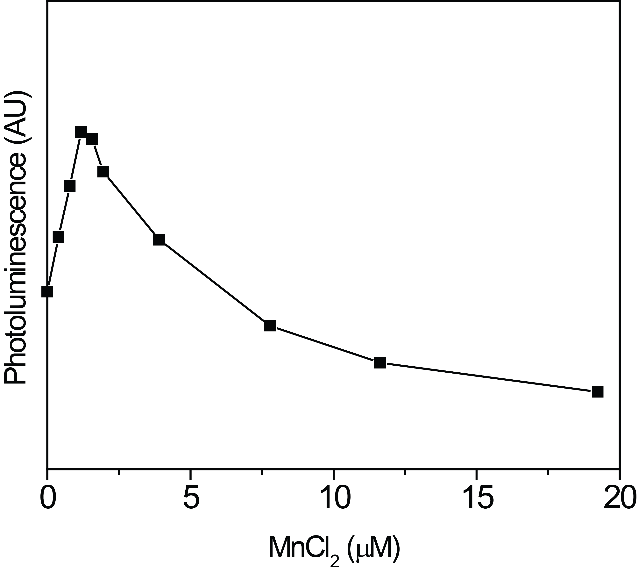


**Figure S9:** Left: Photoluminescence spectra of Mn^2+^:ZnSe/ZnS@SiO_2_ NPs with medium ZnS shells encapsulated in silica suspended in PBS with successive addition of 1 mM MnCl_2_. Right: Corresponding scatter plot.

**Table S1.** *p*-values for thiols and control analytes.

| Analytes | *p*-value |
| --- | --- |
| GSH-DTT | 0.6064518 |
| GLY-DTT | 0.0002633 |
| LYS-DTT | 0.0000016 |
| MnCl_2_-DTT | 0.0000015 |
| NAC-DTT | 0.9267174 |
| NaCl-DTT | 0.0000001 |
| GLY - GSH | 0.0705745 |
| LYS - GSH | 0.0019124 |
| MnCl_2_- GSH | 0.0030278 |
| NAC- GSH | 0.9976071 |
| NaCl- GSH | 0.0002704 |
| LYS - GLY | 0.9137878 |
| MnCl_2_- GLY | 0.9808447 |
| NAC- GLY | 0.0175741 |
| NaCl- GLY | 0.7065187 |
| MnCl_2_- LYS | 0.9997852 |
| NAC- LYS | 0.0003176 |
| NaCl- LYS | 0.9997029 |
| NAC-MnCl_2_ | 0.0004637 |
| NaCl-MnCl_2_ | 0.9847784 |
| NaCl-NAC | 0.0000373 |
